# Tyrant Dinosaur Evolution Tracks the Rise and Fall of Late Cretaceous Oceans

**DOI:** 10.1371/journal.pone.0079420

**Published:** 2013-11-06

**Authors:** Mark A. Loewen, Randall B. Irmis, Joseph J. W. Sertich, Philip J. Currie, Scott D. Sampson

**Affiliations:** 1 Natural History Museum of Utah and Department of Geology & Geophysics, University of Utah, Salt Lake City, Utah, United States of America; 2 Department of Earth Sciences, Denver Museum of Nature & Science, Denver, Colorado, United States of America; 3 Department of Biological Sciences, University of Alberta, Edmonton, Alberta, Canada; Royal Ontario Museum, Canada

## Abstract

The Late Cretaceous (∼95–66 million years ago) western North American landmass of Laramidia displayed heightened non-marine vertebrate diversity and intracontinental regionalism relative to other latest Cretaceous Laurasian ecosystems. Processes generating these patterns during this interval remain poorly understood despite their presumed role in the diversification of many clades. Tyrannosauridae, a clade of large-bodied theropod dinosaurs restricted to the Late Cretaceous of Laramidia and Asia, represents an ideal group for investigating Laramidian patterns of evolution. We use new tyrannosaurid discoveries from Utah—including a new taxon which represents the geologically oldest member of the clade—to investigate the evolution and biogeography of Tyrannosauridae. These data suggest a Laramidian origin for Tyrannosauridae, and implicate sea-level related controls in the isolation, diversification, and dispersal of this and many other Late Cretaceous vertebrate clades.

## Introduction

The isolation of western North America (Laramidia) from eastern North America by the Western Interior Seaway (WIS) at the beginning of the Late Cretaceous (∼95 million years ago) [1] had profound consequences for the evolution of previously widespread Laurasian non-marine faunas. The biotic effects of this long interval of Laramidian isolation are apparent in the abundance and diversity of well-known Campanian-aged vertebrate faunas. Remarkably, observed taxonomic richness of late Campanian Laramidia is significantly higher across many non-marine vertebrate clades than that of Maastrichtian Laramidia [2], [3]. This pattern is unexpected given that the far greater areal extent of North America during the Maastrichtian is predicted to have resulted in higher diversity because of the documented relationship between species richness and area known as the species-area effect [4]. The processes involved in generating and maintaining this heightened Campanian diversity remain controversial and poorly understood.

Campanian Laramidian ecosystems appear to have been characterized by high beta diversity and basin-scale endemism, with evidence for higher-order regional separation into northern and southern provinces [5], [6], [7]. Although these patterns of endemism are now well-recognized [5]–[8], no consensus has emerged regarding the evolutionary mechanisms or tempo that produced these differences. In the absence of recognized physiographic barriers, previous work implicated latitudinal variation in floral diversity linked to latitudinal climate variability, sea-level fluctuations, and/or orogenic activity [5], [7]–[10].

**Figure 1:**
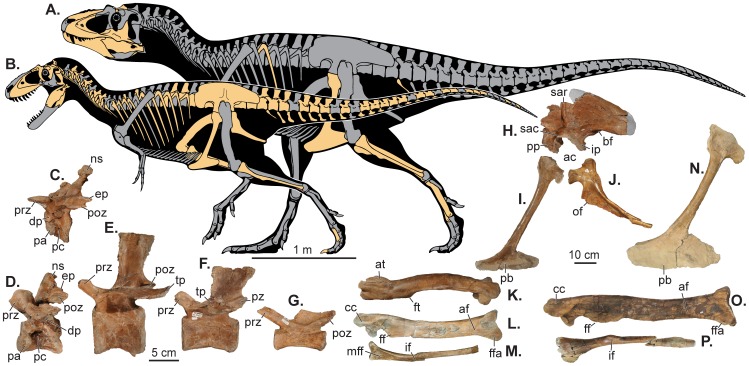
Skeletal reconstructions and postcranial elements of Utah tyrannosaurs. (A) Skeletal outlines showing recovered elements of *Lythronax argestes* (UMNH VP 20200) and (B) *Teratophoneus curriei* (UMNH VP 16690). Selected postcranial elements of *Teratophoneus* in left lateral view: (C) cervical vertebra 3; (D) cervical vertebra 9; (E–G) three caudal vertebrae; (H) right ilium (photoreversed with left illium in the background in grayscale); (I) pubis; (J) ischium; (K) right femur in lateral view; (L) right tibia in anterior view; and (M) right fibula in medial view. Elements of *Lythronax* figured include: (N) the left pubis in lateral view; (O), left tibia in anterior view (photoreversed); and (P) left fibula in medial view (photoreversed). Scale bar for *a* and *b* is 1 meter, *c-g* 5 cm and *h-p* 10 cm. Abbreviations: ac, acetabulum; af, astragalar facet; bf, brevis fossa; cc, cnemial crest; dp, diapophysis; ep, epipophysis; ff, fibular flange; ffa, fibular facet; ft, fourth trochanter; if, iliofibularis muscle scar; ip, ischial peduncle; lt, lesser trochanter; mff, fibular fossa; ns, neural spine; of, obturator flange; pa, parapophysis; pb, pubic boot; pc, pleurocoel; pp, pubic peduncle; poz, postzygapophysis; prz, prezygapophysis; sac, supraacetabular crest; sar, supraacetabular ridge; tp, transverse process.

Clades of carnivorous non-marine taxa provide an excellent independent biogeographic test of these hypotheses because they are presumably less affected by regional floral differences. Among Campanian-Maastrichtian carnivores, tyrannosaurid theropod dinosaurs are one of the best studied and represented clades [11], and are therefore ideal for elucidating the origin and tempo of changes in Laramidian diversity patterns. Recent finds from southern Laramidia (Utah and New Mexico, USA) [12], [13] allow us to test hypotheses of tyrannosaurid diversification in the context of Late Cretaceous climate and sea-level change. Work in the Kaiparowits Basin of southern Utah has recovered abundant new fossils critical for testing such patterns, including the oldest-known tyrannosaurid and the most complete tyrannosaurid specimen discovered from southern Laramidia (Fig. 1). The phylogenetic and biogeographic implications of the new taxon from the Wahweap Formation (∼80 Ma), together with a nearly complete skeleton of the poorly understood *Teratophoneus curriei* [13] from the overlying Kaiparowits Formation (∼76 Ma), are examined in the context of the isolation and dispersal of Laramidian vertebrates during the latest Cretaceous.

## Results

### Systematic Paleontology

Dinosauria Owen 1842 [14] *sensu* Padian and May 1993 [15]

Theropoda Marsh 1881 [16] *sensu* Gauthier 1986 [17]

Coelurosauria Huene 1914 [18] *sensu* Sereno et al. 2005 [19]

Tyrannosauroidea Walker 1964 [20] *sensu* Holtz 2004 [21]

Tyrannosauridae Osborn 1906 [22] *sensu* Sereno et al. 2005 [19]

Tyrannosaurinae Osborn 1906 [22] *sensu* Sereno 2005 [19]

***Lythronax argestes*** Loewen, Irmis, Sertich, Currie, and Sampson 2013 **gen. et sp. nov.** urn:lsid:zoobank.org:act:DE2997BB-1D2B-47C2-A341-80D6FCEFDB34

#### Etymology

*Lythronax*, from *lythron* (Greek), gore, and *anax* (Greek), king; and *argestes* (Greek), the Homeric wind from the southwest, in reference to the geographic location of the specimen within North America.

#### Holotype

**Figure 2:**
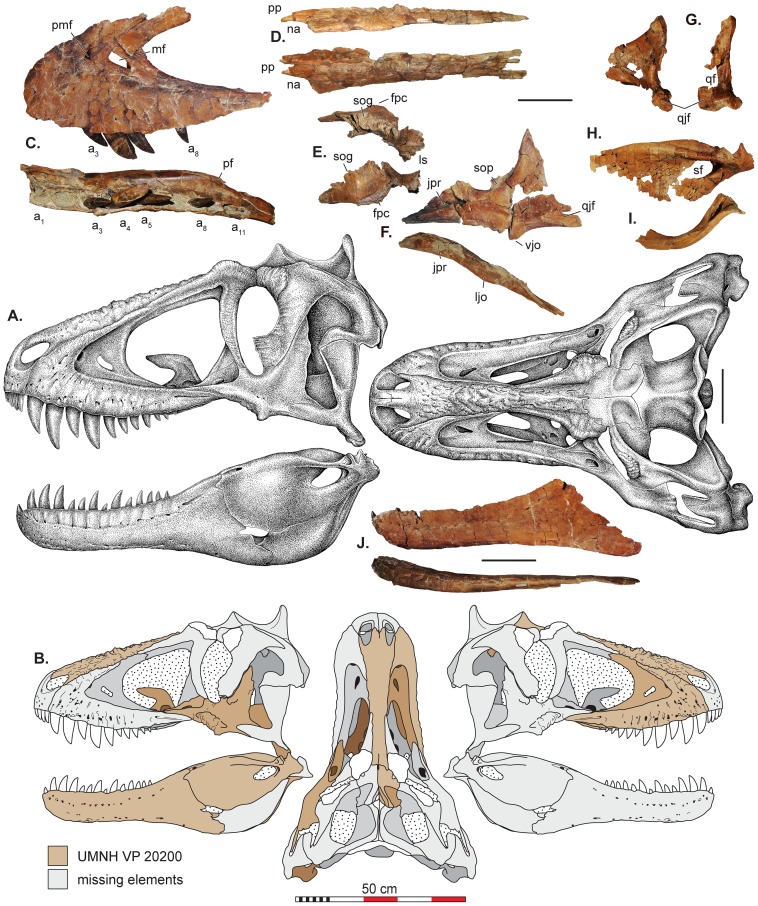
Skull reconstructions and selected cranial elements of *Lythronax argestes*. These stippled reconstructions (A) are based on cranial elements recovered (B) for UMNH VP 20200. Selected elements of *L. argestes* holotype (UMNH VP 20200) including: (C) maxilla in lateral (photoreversed) and ventral views; (D) nasal in left lateral and dorsal view; (E) photoreversed frontal and laterosphenoid in lateral and dorsal view; (F) jugal in left lateral and dorsal view; (G) quadrate in left lateral and caudal view; (G) surangular in left lateral view; (I) prearticular in left lateral view; (J) dentary in lateral and ventral views. Abbreviations: a1-a11, alveoli 1–11; fpc, frontoparietal midsagittal crest; jf, jugal flange of the quadrate; jpr, jugal pneumatic recess; ljo, lateral jugal ornamentation; ls, laterosphenoid; mf, maxillary fenestra; na, naris; pf, palatine flange; pp, premaxillary process of nasal; qf, quadrate foramen; qjf, quadratojugal facet; sf, surangular foramen; sog, supraorbital groove; sop, suborbital process; vjo, ventral jugal ornamentation. Scale bars in A and C–J represent 10 cm and scale bar in B represents a total of 50 cm.

Natural History Museum of Utah (UMNH) VP 20200, an associated partial skeleton including right maxilla, both nasals, right frontal, left jugal, left quadrate, right laterosphenoid, right palatine, left dentary, left splenial, left surangular, left prearticular, a dorsal rib, a caudal chevron, both pubes, left tibia, left fibula, and left metatarsals II and IV (see Figs. 1 and 2). All material is housed at the Natural History Museum of Utah in Salt Lake City, Utah, USA.

#### Type, locality, horizon and age

The holotype was recovered from the lower part of the middle member of the Wahweap Formation in Grand Staircase-Escalante National Monument (GSENM), Kane County, southern Utah, USA. The quarry site, UMNH VP Locality 1501, is located near Nipple Butte, ∼5 m above the contact between the lower and middle members of the Wahweap Formation. This horizon occurs stratigraphically between bentonitic ash beds that are ^40^Ar/^39^Ar radioisotopically dated to 79.9±0.3 Ma and 80.6±0.15 Ma [23], [24], placing *Lythronax* in the middle Campanian.

#### Diagnosis

Tyrannosaurid theropod diagnosed by the following autapomorphies: sigmoidal lateral margin of maxilla; ratio of transverse width of anterior portion of nasal to tranverse width of middle portion greater than 2.5; prefrontal and postorbital contact surfaces on frontal nearly in contact, separated only by very narrow, deep dorsoventral groove; presence of distinct suboccular flange on jugal. *Lythronax* is also diagnosed by the presence of the following unique combination of characters: differs from *Alioramus* and all other tyrannosauroids except *Teratophoneus* and *Bistahieversor* in the presence of 11 maxillary alveoli; differs from *Appalachiosaurus*, *Alioramus* and all other tyrannosauroids except *Bistahieversor, Tyrannosaurus* and *Tarbosaurus* in having a concave lateral margin of dentary and a broad postorbital process of jugal (relative to total jugal length); differs from all other tyrannosauroids except *Tyrannosaurus* and *Tarbosaurus* in having a laterally expanded caudal portion of the skull, such that the orbits are directed rostrodorsally; differs from *Gorgosaurus, Albertosaurus* and *Teratophoneus* in having a large, distally expanded pubic boot (with an anterioposterior length at least 60% of total pubic length); and differs from *Teratophoneus, Bistahieversor, Tyrannosaurus* and *Tarbosaurus* in possessing a dorsally expanded ascending process of the astragalus (with the height of the ascending process greater than the width of the astragalus and calcaneum).

*Teratophoneus curriei* Carr et al. 2011 [13]

#### Material

**Figure 3:**
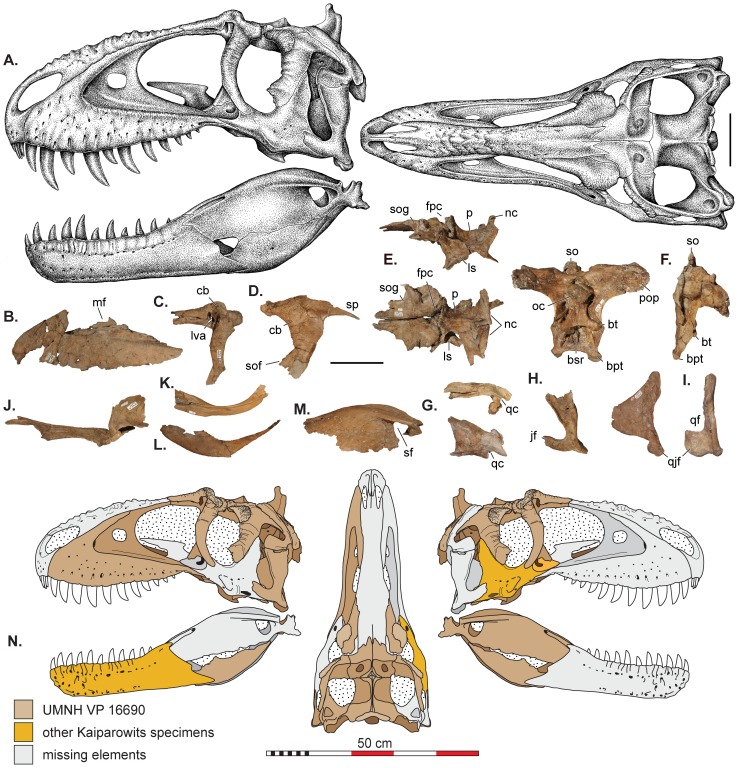
Skull reconstructions and selected cranial elements of *Teratophoneus curriei*. These stippled reconstructions (A) are based on all available material. Some of the preserved elements of the referred specimen *T. curriei* (UMNH VP 16690) including: (B) left maxilla in lateral view, (C) both lacrimals superimposed and in lateral view; (D) photoreversed postorbital in lateral view; (E) frontals, parietals, and laterosphenoids in lateral and dorsal views; (F) braincase in caudal and lateral view; (G) squamosal in lateral and dorsal views; (H) quadratojugal in lateral view; (I) quadrate in lateral and caudal views; (J) left palatine in lateral view; (K) prearticular in left lateral view; (L) angular in left lateral view; (M) surangular in lateral view. Element recovery maps (N) of *T. curriei* (UMNH VP 16690) from which the reconstruction in A are derrived. Other Kaiparowits T. curriei specimens include two right jugals (UMNH VP 16691 & BYU 8120) and a left dentary from BYU 8120. Abbreviations: bpt, basipterygoid process; bt, basal tubera; bsr, basisphenoid recess; cb, cornual boss; fpc, frontoparietal midsagittal crest; jf, jugal flange of the quadrate; ls, laterosphenoid; lva, lacrimal vacuity; mf, maxillary fenestra; nc, nuchal crest; oc, occipital condyle; p, parietal; pop, paroccipital process; qc, quadrate cotylus; qf, quadrate foramen; sf, surangular foramen; so, supraoccipital; sof, suborbital flange; sog, supraorbital groove. All scale bars represent 10 cm except N which represents 50 cm.

The holotype specimen represents a single individual with specimen numbers Brigham Young University (BYU) 8120/9396; BYU 8120/937; BYU 826/9720; BYU 9398; and BYU 13719. This specimen is here collectively referred to as BYU 8120. The newly reported specimen, UMNH VP 16690, is a single associated subadult individual, including most of the skull and axial column, the pelvis and part of the right hind limb (see Figs. 1 and 3). The skull includes the left maxilla, lacrimals, postorbitals, right squamosal, left quadratojugal, left quadrate, frontals, parietals, braincase, ectopterygoids, an epipterygoid, pterygoids, angulars, surangulars, left prearticular, left articular, and multiple teeth. The skull is lacking the premaxillae, right maxilla, nasals, jugals, left squamosal, palatines, vomer, dentaries, splenials and the right prearticular and articular. Axial elements include: atlas, seven postaxial cervical vertebrae; cervical ribs; eight dorsal vertebrae; 14 dorsal ribs; two sacral vertebrae; 34 caudal vertebrae; 19 chevrons. The appendicular skeleton is represented by portions of both ilia, both pubes, both ischia, complete right femur, tibia, fibula, one pedal phalanx, and one ungual. An isolated jugal, UMNH VP 16691, from the Kaiparowits Formation, shares with the jugal from the holotype an autapomorphy of a raised rugosity at the rostralmost point of the articular surface for quadratojugal.

#### Locality, horizon and age

The holotype and referred specimens occur in the middle unit of the upper Campanian Kaiparowits Formation, in GSENM, Kane County, southern Utah, USA. Exact locality data for the holotype BYU 8120 are not available; however, a contemporary newspaper article from 1981 describing the location of the site places the specimen in the middle member of the Kaiparowits Formation in ‘The Blues’ exposures northeast of Henrieville, Utah [25]. UMNH VP 16690, the referred subadult specimen, and the referred jugal UMNH VP 16691, were discovered in the lower middle member of the Kaiparowits Formation in the area of Horse Mountain. UMNH VP 16690 was recovered from UMNH VP Locality 597, approximately 290 meters above the base of the formation, and UMNH VP 16691 was recovered approximately 160 meters above the base of the formation. Exact locality data for UMNH VP specimens are on file at the Natural History Museum of Utah, Salt Lake City, Utah, USA. The chronostratigaphic ages of these specimens are constrained by ^40^Ar/^39^Ar dating of sanidine crystals from bentonite ash beds with ages of 76.46±0.14 Ma from 80 m above the basal contact and 75.51±0.15 Ma from approximately 420 m above the base of the formation [24], [26], placing *Teratophoneus* in the late Campanian.

#### Revised diagnosis

Tyrannosaurid theropod diagnosed by the following autapomorphies: the midpoint of the maxillary fenestra is situated caudal to the midpoint of the space between rostral edge of antorbital fossa and rostral edge of antorbital fenestra; raised rugosity on jugal at rostralmost point of articular surface for quadratojugal. *Teratophoneus* is also diagnosed by the presence of the following unique combination of characters: differs from *Alioramus* and all other tyrannosauroids except *Bistahieversor* and *Lythronax* in presence of 11 maxillary alveoli and raised articular surface with ventral ridge for quadratojugal on jugal; differs from *Lythronax, Tyrannosaurus* and *Tarbosaurus* in having arcuate shape in lateral view of the prefrontal scar on frontal; differs from *Alioramus* and all other tyrannosauroids except *Bistahieversor* in having fronto-parietal midline peak subequal in height to nuchal crest; differs from *Tyrannosaurus* and *Tarbosaurus* in having overlap of rostral end of parietal onto caudal surface of frontal; differs from *Alioramus, Gorgosaurus, Albertosaurus*, the Dinosaur Park Formation tyrannosaurid, *Daspletosaurus* and the Two Medicine Formation tyrannosaurid in having surangular shelf that ventrolaterally overhangs surangular foramen; differs from *Alioramus*, *Gorgosaurus, Albertosaurus*, *Daspletosaurus* and the Two Medicine Formation tyrannosaurid in having cervical neural spines taller than face of centrum; and differs from *Appalachiosaurus, Alioramus*, *Gorgosaurus, Albertosaurus* and *Lythronax* in possessing relatively low ascending process of astragalus (with height of ascending process less than width of astragalus).

### Description and Comparisons

Like other tyrannosaurids (e.g., *Albertosaurus, Gorgosaurus*, *Daspletosaurus*), *Lythronax* and *Teratophoneus* possess a relatively short rostrum (<0.65 total skull length) and broad skull (>0.4 total skull length). Based on the strongly sigmoidal lateral margin of the maxilla and jugal, as well as the mediolateral width of the frontal, the posterior portion of the skull of *Lythronax* was substantially expanded mediolaterally, with anterolaterally directed orbits, features otherwise present only in *Tyrannosaurus* and *Tarbosaurus* among tyrannosaurids. In contrast, the skull of *Teratophoneus* is more similar in overall morphology to those of more basal tyrannosaurids (*Albertosaurus*, *Bistahieversor*, *Daspletosaurus*, and *Gorgosaurus*), with moderately anterolaterally directed orbits and only a modestly expanded posterior skull (Figs. 2 and 3).

As in all known tyrannosaurids, the maxilla of *Lythronax* and *Teratophoneus* is strongly convex ventrally. Similar to *Bistahieversor* (11), *Tarbosaurus* (12–13) and *Tyrannosaurus* (12–13), a reduced number of maxillary alveoli (11) are present relative to other tyrannosaurids such as *Albertosaurus* (14–16), *Daspletosaurus* (15–16), and *Gorgosaurus* (14–15). The maxillary dentition of *Lythronax* is also notably heterodont; the first five teeth are much larger than the remaining posterior six teeth. In overall morphology, the maxilla of *Lythronax* is notably robust and sigmoidal in lateral contour, with a well-developed palatal shelf comparable to that of *Tyrannosaurus*. The jugal of *Lythronax* is similarly robust, with a sinusoidal lateral profile and wide postorbital process. A strongly developed process on the anterior border of the postorbital process indicates the presence of a large subocular flange, contrasting with the much more modest postorbital subocular flange of *Teratophoneus* and other tyrannosaurids.

The dentary ramus of *Lythronax* is strongly concave laterally, mirroring the sigmoidal contour of the maxilla and extreme posterior expansion of the skull. The posterior end of the dentary is also dorsoventrally flared, indicating a deep post-dentary region, as in *Tarbosaurus* and *Tyrannosaurus*, and unlike the condition in *Albertosaurus, Daspletosaurus*, and *Gorgosaurus*. The surangulars of both *Lythronax* and *Teratophoneus* display well-developed, dorsoventrally-deep shelves similar to those of other tyrannosaurids. *Lythronax* further resembles *Tyrannosaurus* in possessing a dorsally concave surangular shelf.

The postcrania of both *Lythronax* and *Teratophoneus* are similar in overall morphology to those of other tyrannosaurids. Tyrannosaurid synapomorphies present in both taxa include the presence of a large anterior process on the distal pubic boot, and a deeply excavated medial fossa on proximal end of the fibula. The pubis of *Lythronax* is notable for its dorso-ventrally expanded pubic boot, proportionally most similar to those of *Tarbosaurus* and *Tyrannosaurus*, and contrasting with the less-expanded condition in taxa such as *Albertosaurus*, *Daspletosaurus*, and *Gorgosaurus*.

## Discussion

**Figure 4:**
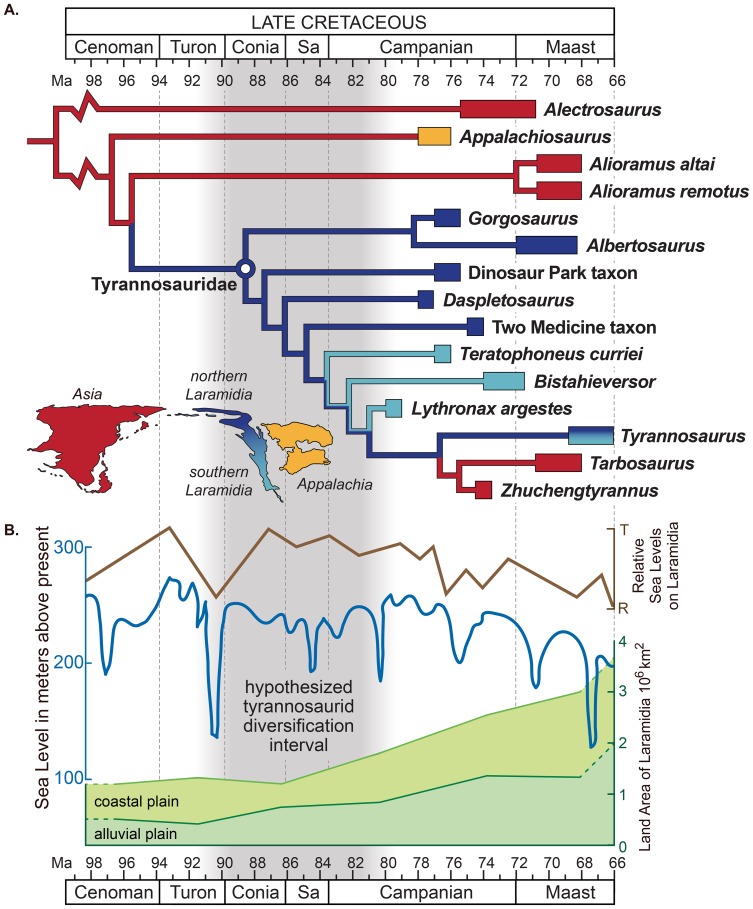
Sea level change and the hypothesized evolutionary diversification of Tyrannosauroidea. Sea level indicators include: (A) Time-calibrated phylogenetic relationships and paleobiogeographic distribution of tyrannosaurids with biogeographic origin indicated by color (see Methods and File S1 for details of analyses and the stratigraphic and phylogenetic relationships of tyrannosauroids within theropod dinosaurs). (B) Late Cretaceous regional transgression-regression cycles on Laramidia (brown [31]); global sea-level fluctuations (blue [34], [35]); and areal extent of Laramidia at minimum (alluvial plain) and maximum (alluvial plain and coastal plain) sea levels (green [1]).

Our comprehensive phylogenetic analysis (see Methods and File S1) recovers the Campanian taxa *Lythronax, Bistahieversor*, and *Teratophoneus* as successive sister groups to the Maastrichtian clade of *Tarbosaurus* and *Tyrannosaurus* (see Fig. 4). This southern Laramidian radiation is more highly-nested within Tyrannosauridae than in other previously recovered topologies [11]–[13], [27] and is more closely allied with *Tarbosaurus* and *Tyrannosaurus* than any of these taxa are to *Daspletosaurus*.

The new placement of these southern taxa is extremely well-supported in our analysis, and robust to taxon selection (see Methods and File S1). Relative to more basal northern taxa (*Albertosaurus*, *Daspletosaurus*, *Gorgosaurus*), this clade is characterized by a reduced number of maxillary teeth (<13) and relatively shorter, broader skulls with transversely expanded postorbital regions. This pushes back the origin of the *Tyrannosaurus*-style skull morphology or rostrally-oriented orbits and expanded postorbital regions to at least 80 Ma, implying the continuous presence of two distinct cranial morphologies in Laramidian tyrannosaurids for at least 10 million years. Several other Campanian–Maastrichtian tyrannosauroid taxa—the Appalachian forms *Appalachiosaurus* [28] and *Dryptosaurus* [29], and the Asian taxon *Alioramus* [27]—are recovered outside of Tyrannosauridae. To date, the only known non-Laramidian tyrannosaurids are *Tarbosaurus* and *Zhuchengtyrannus* [30], both from the latest Cretaceous of Asia.

The highly-nested phylogenetic position of *Lythronax*, combined with its stratigraphic position as the oldest tyrannosaurid, implies that the origin and initial diversification of Tyrannosauridae occurred prior to 80 Ma, a minimum of two million years earlier than previously thought. More importantly, it implies that the major groups of tyrannosaurids (i.e., albertosaurines and tyrannosaurines) had diversified prior to 80 Ma, rather than during the late Campanian (75–70 Ma). On Laramidia, widespread lowland areas between the WIS and Sevier orogenic belt were not exposed until the earliest Campanian (∼83 Ma) [1]. Similarly, the documented presence of late-surviving basal tyrannosauroids in Asia (*Alectrosaurus*, *Alioramus*, *Bagaaratan*) and on Appalachia (*Appalachiosaurus* and *Dryptosaurus*) most likely reflects the abundance of Campanian-Maastrichtian dinosaur-bearing sediments, whereas fossiliferous Turonian-Santonian non-marine intervals are poorly exposed [1], [2] and understudied. Given the absence of tyrannosaurids in extensive lower and middle Campanian dinosaur-bearing exposures outside of Laramidia [28], [29], current evidence suggests a Laramidian origin for Tyrannosauridae during the late Turonian to earliest Campanian (∼90–82 Ma), an interval of high global sea-levels when the WIS was at its greatest extent [1], [31]. We propose that the radiation post-dates the latest Turonian, because a major regression at ∼91–90 Ma (Fig. 4) would have allowed a more cosmopolitan distribution of Tyrannosauridae.

Within Laramidia, tyrannosaurids exhibited distinct distributional patterns during the Campanian. Like other dinosaur clades [5]–[7], each depositional basin contains endemic species, but phylogenetic relationships of these taxa indicate higher-level biogeographic divisions between northern (Wyoming, Montana, and Canada) and southern (Utah and New Mexico) Laramidia [5]–[8], [32]. To test these hypotheses of regionalism, origin, cladogenesis, and dispersal, we conducted a phylogenetic biogeographic analysis of Tyrannosauridae using a temporally-calibrated phylogeny and a likelihood model of dispersal, extinction, and cladogenesis [33]. Our reconstructions, including sensitivity analyses (see Methods), provide unambiguous support for a Laramidian ancestral range of Tyrannosauridae. They further robustly support the *Teratophoneus* lineage as a southern dispersal event, and a widespread Laramidian ancestral range for the clade of *Bistahieversor*, *Lythronax*, *Tarbosaurus*, and *Tyrannosaurus*, with allopatric speciation events between northern and southern Laramidia leading to the origination of *Bistahieversor*, *Lythronax*, and *Tarbosaurus* + *Tyrannosaurus*. Finally, these analyses suggest that the lineage leading to *Tarbosaurus* and *Tyrannosaurus* had a northern Laramidian ancestral range, with the lineage leading to *Tarbosaurus* (and *Zhuchengtyrannus*) the result of dispersal to Asia.

Current data suggest that early tyrannosauroid dinosaurs achieved a widespread Laurasian distribution by the Late Jurassic [11], a pattern that persisted through the middle Cretaceous. Rising sea levels and the formation of the WIS between 100 and 95 Ma isolated Laramidia from Appalachia and Asia; geologic evidence indicates this at times separated individual non-marine depositional basins within Laramidia, and the Sevier orogeny provided a major biogeographic barrier to the west [1], [9]. We hypothesize that the full isolation of Laramidia coincided with the origin of Tyrannosauridae, and that separation of individual basins by seaway incursion caused much of the initial diversification within the clade. Only with the lowering of sea levels towards the end of the Campanian or beginning of the Maastrichtian [1], [31], [34], [35] did tyrannosaurids disperse into Asia, as represented by *Tarbosaurus* and *Zhuchengtyrannus*. Similarly, retreat of the WIS may also have been linked to widespread dispersal of *Tyrannosaurus rex* [36].

These patterns of tyrannosaurid evolution mirror those of other large non-marine vertebrate clades from Laramidia [5]–[8]. Ceratopsian and hadrosauroid dinosaurs, crocodylians, and baenid turtles all show a similar phylogenetic separation between widespread basal forms that diverged prior to 90 million years ago, and predominantly Laramidian highly-nested forms from the Campanian-Maastrichtian, [5]–[8], [37]–[39]. Several of these clades also show evidence for latest Cretaceous dispersal to Asia (e.g., *Charonosaurus* [40], *Olorotitan* and *Saurolophus* [41], and *Sinoceratops* [42]), and single species that are widespread in the late Maastrichtian of Laramidia (e.g., *Edmontosaurus* and *Triceratops*). In a pattern similar to that of Tyrannosauridae, alligatoroid crocodylians and baenid turtles show some evidence of southern Laramidian Campanian taxa whose lineages appear later during the Maastrichtian in northern Laramidia [43], [44].

These concordant patterns across disparate non-marine vertebrate clades suggest a common cause, which we hypothesize to be transgression and regression cycles of the WIS, isolating these clades on Laramidia. Rather than generating diversity through anagenetic change within each basin [10], or through strictly orogenic processes [9], we propose that seaway transgressions isolated non-marine sedimentary basins and promoted allopatric diversification, the results of which are recorded in early and middle Campanian deposits throughout Laramidia, and are supported by our biogeographic analysis. As the WIS regressed, some lineages—including tyrannosaurids, ceratopsians, and hadrosaurids—dispersed to eastern Asia, and some members of these groups became more widespread throughout Laramidia (e.g., *Edmontosaurus*, *Triceratops* and *Tyrannosaurus*). The apparent influence of sea level on both intra- and intercontinental scale non-marine vertebrate distribution during the Campanian-Maastrichtian of Laramidia had profound implications for the evolution of terrestrial ecosystems during this time, leading to disparate Late Cretaceous faunas across adjacent landmasses.

## Materials and Methods

### Paleontological Ethics Statements

All of the specimens described in this paper (UMNH VP 16690, UMNH VP 16691, and UMNH VP 20200) are permanently reposited in the collections of the Natural History Museum of Utah, 301 Wakara Way, Salt Lake City, Utah, USA. Locality information is available from the registrar of the museum as per museum policy. All necessary permits were obtained for the described study, which complied with all relevant regulations. All of these specimens were collected under permits obtained from the United States Department of the Interior's Bureau of Land Management (BLM) for work conducted in the BLM-administered Grand Staircase-Escalante National Monument.

### Nomenclatural Acts

The electronic edition of this article conforms to the requirements of the amended International Code of Zoological Nomenclature, and hence the new names contained herein are available under that Code from the electronic edition of this article. This published work and the nomenclatural acts it contains have been registered in ZooBank, the online registration system for the ICZN. The ZooBank LSIDs (Life Science Identifiers) can be resolved and the associated information viewed through any standard web browser by appending the LSID to the prefix “http://zoobank.org/”. The LSID for this publication is: urn:lsid:zoobank.org:pub:B4EC9A4F-B7F3-4DF4-9C85-B6FE790B9A91. The LSID for *Lythronax argestes* is: urn:lsid:zoobank.org:act:DE2997BB-1D2B-47C2-A341-80D6FCEFDB34. The electronic edition of this work was published in a journal with an ISSN (1932-6203), and has been archived and is available from the following digital repositories: LOCKSS (http://www.lockss.org); PubMed Central (http://www.ncbi.nlm.nih.gov/pmc).

### Institutional Abbreviations

See Table S1 in File S1.

### Skull Reconstruction of Lythronax

**Figure 5:**
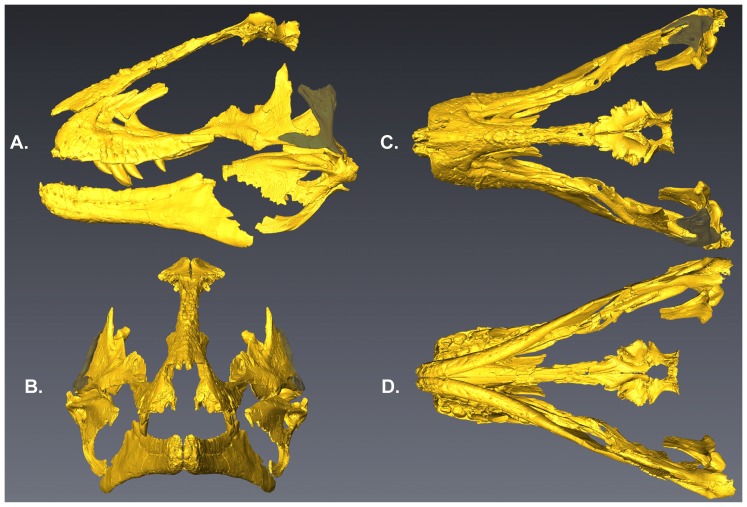
Reconstruction of the skull of *Lythranax* (UMNH VP 20200). Elements were surface scanned and then digitally mirrored and expressed as a 3-D surface scan in lateral (A), rostral (B), dorsal (C) and ventral (D) views. The quadratojugal, shaded gray, is that of *Teratophoneus* scaled to fit the larger skull of *Lythronax*. The quadratojugal is conservative across Tyrannosauridae and was used to place the quadrate.

The skull reconstructions of *Lythronax* in figure 2 are derived from surface scans of the elements mirrored and arranged in 3-dimensional space digitally. Figure 5 shows the configuration of the skull in multiple views using this method. The quadratojugal of *Teratophoneus* was scaled to size and placed in the reconstruction to constrain the probable placement of the quadrate of *Lythronax* as the quadratojugal is a highly conservative element with respect to shape within Tyrannosauridae.

### Phylogenetic Analysis

In order to formulate hypotheses of the phylogenetic relationships of *Lythronax argestes* and *Teratophoneus curriei* relative to other tyrannosauroid theropod dinosaurs, a phylogenetic analysis using cladistic parsimony was employed. The analysis comprised 54 taxa and 501 characters (303 cranial and 198 postcranial). See File S1 for sources of character scoring, characters, and taxon scorings (Table S3 in File S1) Numerous features (e.g., cranial and hindlimb features) indicate that the two taxa were members of the clade Tyrannosauroidea; therefore selection of ingroup taxa and characters focused on this clade. All valid described tyrannosauroid species, and several currently unnamed taxa, were included in the analysis, for a total of 26 tyrannosauroids. To ensure proper character polarization and determine the position of Tyrannosauroidea among Theropoda, we sampled widely across both coelurosaur and non-coelurosaur neotheropods. The basal theropod *Tawa hallae* was constrained as the outgroup because it is a proximate sister group of Neotheropoda and is known from nearly complete remains [45]. Specimen numbers, institutional abbreviations, and literature sources for each taxon are reported in File S1. Characters used in the analysis (see File S1) were partially derived from previous phylogenies [11], [12], [27], [46]–[49]. In addition to adding a number of new characters and character states, close attention was paid to existing character definitions; a significant number of characters from previous analyses were deleted, combined, or otherwise revised with an eye towards improving clarity and anatomical precision.

**Figure 6:**
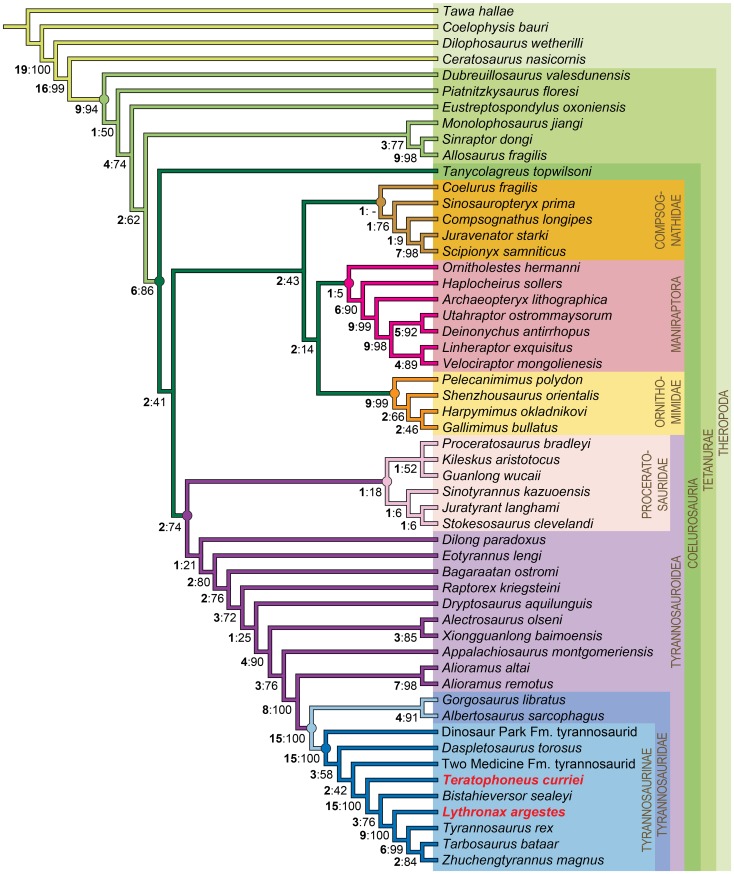
The relationships of *Lythranax argestes* (UMNH VP 20200) and *Teratophoneus curriei* within the larger context of Theropoda. Strict consensus of the two most parsimonious trees with a tree length of 1762 steps for 54 taxa and 501 morphological characters. Bold numbers on the nodes indicate Bremer support indices and GC values are listed to the right of the Bremer support indices. Consistency Index (CI)  = 0.378, Retention Index of (RI)  = 0.797.

The original character-taxon matrix was assembled in Microsoft Excel (Office Professional 2010) imported into Mesquite v.2.75 [50] and is freely available on Morphobank [51] as project 998. The final dataset was analyzed using TNT v. 1.1 [52], [53]. Tree searching followed the parsimony criterion implemented under the heuristic search option using tree bisection and reconnection (TBR) with 10,000 random addition sequence replicates. Zero length branches were collapsed if they lacked support under any of the most parsimonious reconstructions. All characters were equally weighted. Characters 1, 2, 13, 39, 43, 46, 70, 94, 96, 100, 102, 108, 110, 126, 137, 147, 149, 152, 155, 160, 167, 177, 188, 200, 202, 207, 225, 269, 270, 272, 274, 276, 281, 291, 329, 351, 364, 406, 420, 425, 432, 442, 465, 470, 471, 489, and 491 represent nested sets of homologies and/or entail presence and absence information. These characters were set as additive (also marked as ORDERED in highlighted bold text following character description (see File S1)). Two most-parsimonious trees were found; we report the strict consensus of these trees here (Fig. 6). Tree statistics were calculated using TNT. Bootstrap proportions were calculated using 10,000 bootstrap replicates with 10 random addition sequence replicates for each bootstrap replicate [54], [55]. Bremer support was calculated using negative constraints as employed by the BREMER.RUN script supplied with TNT. Character state changes were optimized and synapomorphies for clades (each node is numbered in Figure S1 in File S1) are listed in Table S4 in File S1.

#### Extended Summary of the Results of the Phylogenetic Analysis

We use Holtz's [21] definition of the clade Tyrannosauroidea, defined as the most inclusive clade containing *Tyrannosaurus rex* but not *Ornithomimus edmontonicus*, *Troodon formosus*, or *Velociraptor mongoliensis*. Our usage of Proceratosauridae follows Rauhut et al. [56] as all theropods that are more closely related to *Proceratosaurus bradleyi* than to *Tyrannosaurus rex*, *Allosaurus fragilis*, *Compsognathus longipes*, *Coelurus fragilis*, *Ornithomimus edmontonicus*, or *Deinonychus antirrhopus*. Tyrannosauridae is defined as the least inclusive clade containing *Tyrannosaurus rex*, *Gorgosaurus libratus*, and *Albertosaurus sarcophagus* [19]. Tyrannosaurinae is defined as most inclusive clade containing *Tyrannosaurus rex* but not *Gorgosaurus libratus*, *Albertosaurus sarcophagus* [19].

We recover a monyphyletic Proceratosauridae with two sub-clades (Fig. 6). One clade includes *Stokesosaurus, Juratyrant*, and *Sinotyrannus* and the other is a polytomy including *Proceratosaurus, Kileskus*, and *Guanlong*. Non-proceratosaurid tyrannosauroids closer to tyranosaurids include *Dilong, Eotyrannus, Bagaraatan, Raptorex*, and *Dryptosaurus* in a pectinate array below a clade including *Alectrosaurus* and *Xionguanlong*. We recover and here recognize *Raptorex* as a distinct taxon (see Tsuihiji et al., [57]); regardless of its ontogenetic status, it is morphologically distinct from *Alioramus* or *Tarbosaurus* (cf. Fowler et al., [58]). *Appalachiosaurus* is nested above *Alectrosaurus* and *Xionguanlon*, and a clade of the Asian non-tyrannosaurids *Alioramus altai* and *Alioramus remotus*, is the sister taxon to Tyrannosauridae.

*Daspletosaurus torosus* and unnamed taxa from the Dinosaur Park and Two Medicine formations that were once referred to *Daspletosaurus* sp. are recovered as a paraphyletic assemblage rather than a monophyletic group. Further analysis of these specimens and better stratigraphic data is needed to further resolve these three OTUs, we suspect that the Dinosaur Park Formation taxon may represent *D. torosus*, but regard the Two Medicine Formation taxon as distinct from *D. torosus*. Our analysis recovers *Lythronax argestes* and *Teratophoneus curriei* as tyrannosaurine tyrannosaurids. In an alternate analysis, we ran the type (BYU 8120) and referred specimen (UMNH VP 16690) of *Teratophoneus* as separate OTUs in the analysis and recovered the same position for *Teratophoneus*. Furthermore the deletion of *Teratophoneus* does not change the topology of the tree in any way, with the Two Medicine taxon, *Bistahieversor, Lythronax, Tyrannosaurus, Tarbosaurus*, and *Zhuchengtyrannus* all nested above *Daspletosaurus* as tyrannosaurines. The deletion of *Lythronax* also does not change the topology of the tree, with the Two Medicine taxon, *Teratophoneus, Bhistahieversor, Tyrannosaurus, Tarbosaurus*, and *Zhuchengtyrannus* all nested above *Daspletosaurus* as tyrannosaurines. Deletion of both *Lythronax argestes* and *Teratophoneus curriei* does not affect the position of *Bistahieversor* as a tyrannosaurid, contrary to recent topologies recovered by others [11]–[13], [27].

We also conducted several constraint analyses to test the robusticity of our phylogenetic results relative to the differences with other recent analyses [11]–[13], [27]. To place *Bistahieversor* outside Tyrannosauridae required 67 additional steps. Placing *Alioramus* inside of Tyrannosauridae required 31 additional steps. To recover a tree with both *Bistahieversor* outside of Tyrannosauridae and *Alioramus* inside of Tyrannosauridae required 91 additional steps. Thus, we conclude that the placement of these two taxa within our phylogeny is robust given our dataset.

### Biogeographic Analysis

The primary goal of biogeographic analysis was to infer the ancestral ranges within Tyrannosauridae and among its close outgroups. One area of particular interest was in the spine of the tyrannosaurid tree, and its relationship to northern and southern Laramidian taxa. Because these processes could potentially be dominated by dispersal rather than vicariance, we applied the Dispersal-Extinction-Cladogenesis (DEC) model [33], [59]. Not only was this likelihood method developed to specifically reconstruct ancestral ranges, but it also allows explicit incorporation of geologic ages of nodes through temporally-calibrated branch lengths, rather than using cruder time slicing methods.

#### Taxon Sampling

The phylogenetic analysis discussed above served as the basis for our biogeographic investigations. Because our interest was patterns within Tyrannosauridae and its proximate outgroups, we included in the analysis all described Cretaceous tyrannosauroid taxa except the *Yutyrannus* (which was undescribed at the time). This ensures deep enough sampling to ensure proper area reconstruction at the nodes of interest, but excludes the earliest-diverging tyrannosauroid lineages that extend back into the Jurassic, when paleogeography differed significantly from the Late Cretaceous. This portion of the tree had identical topologies across all recovered most parsimonious trees. *Zhuchengtyrannus* was pruned from the tree because of the uncertainty regarding its geologic age. Hone et al. [30] cite Liu et al. [60] who constrain the top of the formation in which *Zuchengtyrannus* is found at 73.5 Ma. Liu et al. [60] do not include any radioisotopic data to substantiate this claim, nor cite any papers to support it. The paper suggests the formation is somewhere between 120 Ma and 73.5 Ma, but again, without any geochronologic details to substantiate such a claim. Hone et al. [30] acknowledge the tenuous nature of the date. The other taxa found in this formation include: *Shantungosaurus*, a hadrosaurid most closely related to *Edmontosaurus* [37]; *Sinoceratops*, a ceratopsid closely related to *Centrosaurus* [42]; and *Zhuchengceratops*, a leptoceratopsid closely related to *Leptoceratops* [61]. All of these taxa suggest a late Campanian to Maastrichtian age. We omit *Zuchengtyrannus* from the biogeographic analysis because we have no confidence in the chronostratigraphic age of the formation, and because it is the sister taxon to *Tarbosaurus* which is dated to 70.6 Ma. Inclusion of *Zuchengtyrannus* does not affect the results of the biogeographic analysis because it is sister taxon to *Tarbosaurus*, also from Asia, and does not appreciably affect the branch lengths of the phylogeny used for the analysis.

#### Taxon Ages and Temporal Calibration of Trees

Incorporating temporal information directly into the biogeographic analysis requires point ages. Yet, even fossils associated with the most precise radioisotopic ages still have some level of uncertainty. Thus, for each taxon, the midpoint of uncertainty/stratigraphic range was taken as a point estimate. Where possible, the ages were constrained with available radioisotopic ages and other geochronologic data [23], [24], [26] (see Table S5 in File S1). When the age of a taxon was only constrained to a geologic stage and/or other relative age, these were converted to absolute ages using the 2012 Geologic Timescale [62].

The geologic ages of each taxon were used to temporally calibrate the phylogenetic tree by constraining the length of each subtending branch. Because some branches remain unconstrained by available geologic ages at the tips, as in other recent studies [47], [63] we took two approaches to dealing with this temporal uncertainty. In the first approach, this uncertainty was “smoothed” evenly across unconstrained branches, following the method of Ruta et al. [64], Brusatte et al. [65], Nesbitt et al. [45], and Irmis [63]. In the second “strict” approach, we took the fossil record at face value, and assigned a minimal length of 0.1 Ma to unconstrained branches, which results in a long branch at the base of a series of divergences concentrated in a very short interval. We view these temporal calibrations as end-members of the actual tempo of evolution for any clade, which probably includes a combination of gradual and punctuated evolution. Nonetheless, they bracket the uncertainty in temporal calibration given that we expect the true branch lengths to fall somewhere in between.

#### Areas

Our analyses focused on understanding the biogeographic relationships of Tyrannosauridae among North American regions, as well as possible dispersal events to/from Asia. Therefore, we defined three areas within North America (northern Laramidia, southern Laramidia, and Appalachia); Appalachia is naturally separated from Laramidia by the Western Interior Seaway. There is a clear spatial break between Late Cretaceous Laramidian localities in the north (Canada, Montana, Wyoming, North and South Dakota) and those in the south (Utah, New Mexico, Texas). We also included Europe as an area because of the presence of one European tyrannosauroid in the analysis (*Eotyrannus*).

#### Analytical Details

The Dispersal-Extinction-Cladogenesis model was implemented using the software package *Lagrange* (release 20110117). Scripts were assembled using the *Lagrange* web configurator (http://www.reelab.net/lagrange/configurator/index). Like previous analyses [45], reconstructed ancestral widespread ranges were limited to no more than two areas for two reasons: 1) only one taxon has an observed range in more than one area (*Tyrannosaurus rex*), and this is the most phylogenetically-nested and geologically youngest taxon in the analysis, present in a pair of adjacent areas; and 2) phylogenetic biogeographic analyses, including DEC, are biased towards reconstructing widespread ancestral areas at deeper nodes of the tree (see discussion in Nesbitt et al. [45] SOM, and references cited therein).

For all analyses, we employed some conservative constraints on possible direct dispersal routes. Direct dispersal between northern Laramidia-Europe, southern Laramidia-Europe, and southern Laramidia-Asia was disallowed, because any dispersal between these non-adjacent areas would require intermediate dispersal through a third area. For each temporal calibration method, we conducted two analyses. The first analysis (“no weighting”) considered all allowed dispersal routes to be equally likely. The second (“weighted”) assigned a penalty of “1” for dispersal to adjacent areas within the same continent, a penalty of “2” for dispersal across continents, and a penalty of “3” for direct dispersal between Asia and Appalachia, as it would require a less likely and longer distance Arctic dispersal route.

#### Extended Summary of the Results of the Biogeographic Analysis

**Figure 7:**
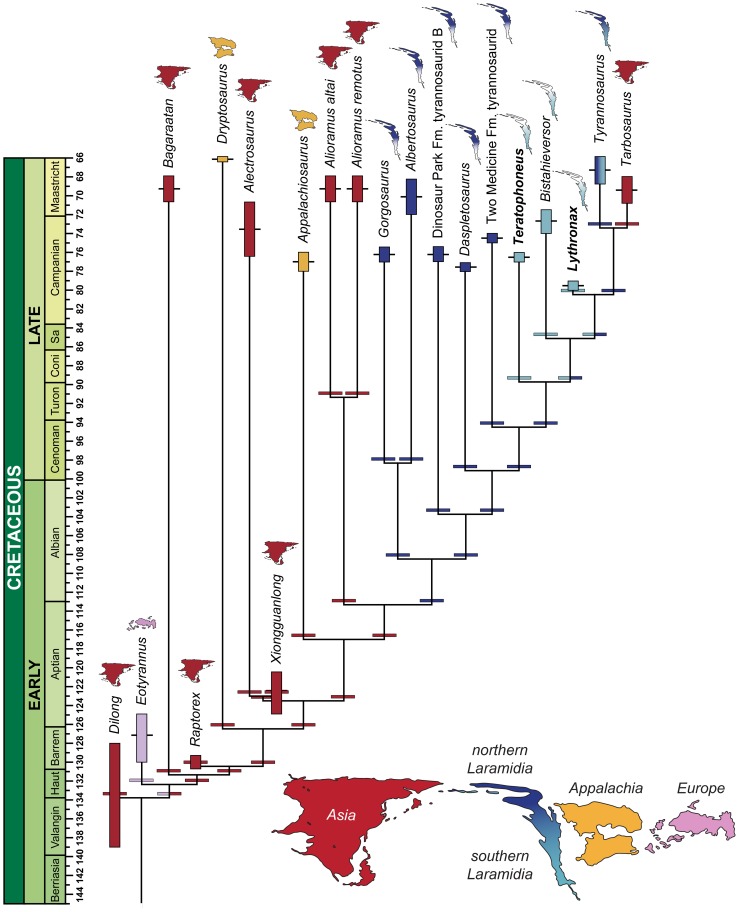
Ancestral range reconstructions from the Dispersal-Extinction-Cladogenesis analyses using a smoothed temporal calibration of the phylogeny. Rectangles indicate the stratigraphic ranges and associated uncertainty, black bars are the midpoint of these ranges, and wide bars represent the reconstructed ancestral range with the highest relative probability. Both the weighted and unweighted analysis results are shown here, because their highest relative probability results for each branch were the same. See Table S6 in File S1 for full results.

**Figure 8:**
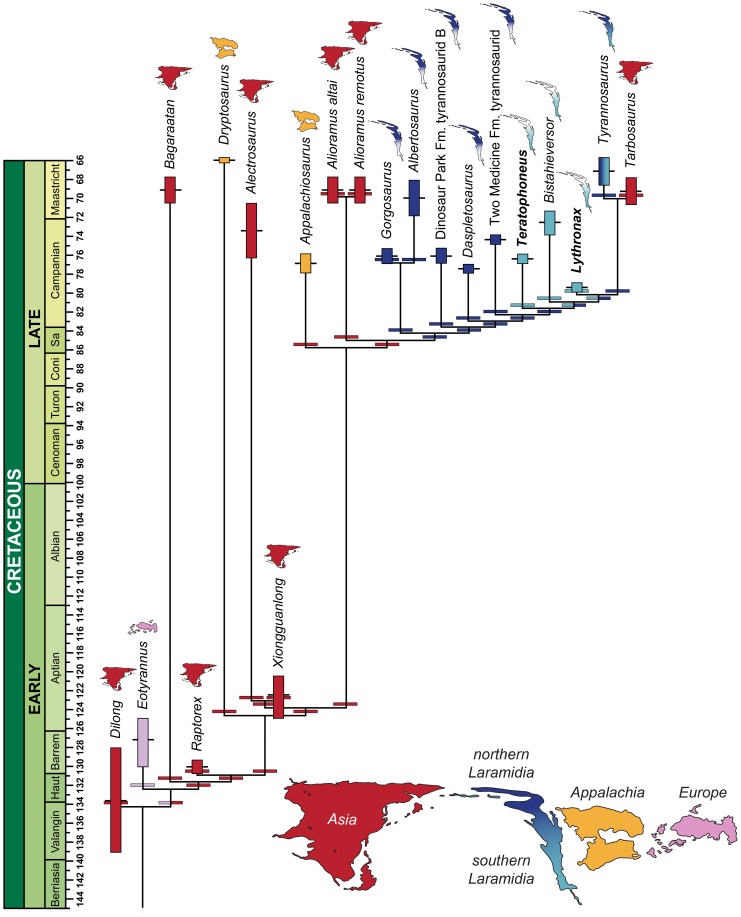
Ancestral range reconstructions from the Dispersal-Extinction-Cladogenesis analyses using a strict temporal calibration of the phylogeny. The exact temporal calibration displayed here is only for display purposes (i.e., some branches have been slightly lengthened for clarity). Rectangles indicate the stratigraphic ranges and associated uncertainty, black bars are the midpoint of these ranges, and wide bars represent the reconstructed ancestral range with the highest relative probability. Both the weighted and unweighted analysis results are shown here, because their highest relative probability results for each branch were the same. See Table S6 in File S1 for full results.

A comprehensive summary of our results is reported in Table S8 in File S1. We also graphically represent the results with the highest relative probability for each analysis in Figs. 7 and 8. We only report results within two log likelihood units of the most likely result for each node; this is the accepted limit for assessing significance [66]. In the figures below, each area is represented by a color; reconstructed ancestral area transformations are shown below each node and along terminal branches leading to OTUs.

All analyses reconstruct either Asia or some combination of Asia, northern Laramidia, and Appalachia ancestral areas for throughout the Cretaceous basal tyrannosauroid stem. Among all analyses, the reconstructions with the highest relative probability suggest that *Appalachiosaurus* had either an Asian or Appalachian ancestral range. The base of Tyrannosauridae is unambiguously reconstructed as having a northern Laramidian ancestral range, and this is true for much of the spine of the tree within basal Tyrannosauridae. Within the “southern clade” of Tyrannosauridae (*Teratophoneus*, *Bistahieversor*, *Lythronax*, *Tarbosaurus*, and *Tyrannosaurus*), Teratophoneus is reconstructed as a dispersal event from northern Laramidia, and the subtending branches for *Bistahieversor* and *Lythronax* are reconstructed as having a widespread Laramidian ancestral range. The branch leading to *Tyrannosaurus* + *Tarbosaurus* is unambiguously reconstructed with a northern Laramidian ancestral range, and *Tarbosaurus* is reconstructed as an Asian dispersal event from a northern Laramidian ancestral area.

## Supporting Information

File S1 Contains the files: Table S1: Institutional Abbreviations; Table S2: Sources of Character Scoring Phylogenetic Analysis Characters; Table S3: Taxon Scorings; Figure S1: Numbered Nodes for the Synapomorphy List; Table S4: Synapomorphy List; Table S5: Stratigraphic Position of Select Taxa; Table S6. Results of the Biogeographic Analysis.
(PDF)
